# Increased angiogenesis by the rotational muscle flap is crucial for nerve regeneration

**DOI:** 10.1371/journal.pone.0217402

**Published:** 2019-06-10

**Authors:** Hung-Chuan Pan, Ming-Hong Chang, Meei-Ling Sheu, Chun-Jung Chen, Jason Sheehan

**Affiliations:** 1 Faculty of Medicine, School of Medicine, National Yang-Ming University, Taipei, Taiwan; 2 Department of Neurological Institute, Taichung Veterans General Hospital, Taichung, Taiwan; 3 Department of Medical Research, Taichung Veterans General Hospital, Taichung, Taiwan; 4 Institute of Biomedical Sciences, National Chung-Hsing University, Taichung, Taiwan; 5 Department of Neurosurgery, University of Virginia, Charlottesville, Virginia, United States of America; Medical University Innsbruck, AUSTRIA

## Abstract

**Background:**

The gold standard surgical treatment of nerve injury includes direct repair, nerve graft, and neurolysis. The underlying effects (either beneficial or detrimental) of angiogenesis during nerve regeneration by rotational muscle flap have not yet determined. We assess the neurological outcome and angiogenesis of nerve injury following a rotational muscle flap.

**Methods:**

We retrospectively analyzed the outcome of the patients with severe radial nerve injury by neurolysis and rotational muscle flap; we also mimicked the clinical situation by nerve crush followed by rotational muscle flap in animals to assess associated angiogenesis factor expression.

**Results:**

Twenty-three out of 25 (92%) cases of severe radial nerve injury underwent neurolysis assisted by muscle flap rotation and eventually reached their preinjury neurological outcome. In the animal study, both FITC–dextran and Dil infusion showed a remarkably increased vascular structure in the crushed nerve integrated by the muscle flap and abolished by Avastin injection. The rotational muscle flap significantly increased angiogenesis factor expression, and this was attenuated by Avastin injection. The increased angiogenesis factor expression paralleled the improvement seen in neurobehavioral and electrophysiological studies as well as the significant expression of nerve regeneration markers and the restoration of denervated muscle morphology.

**Conclusion:**

Based on the clinical and animal data analysis, we conclude that muscle flap rotation provides a platform for angiogenesis in the acceleration of nerve regeneration. It appears that the muscle flap rotation augmented the nerve regeneration process which may be beneficial for nerve repair in clinical application.

## Introduction

The gold standard treatment of peripheral nerve injury is to restore the preinjury function of the damaged nerve and to improve quality of life [1]. The optimal surgical approach depends on the pathology of the nerve injury per sec. Primary repair is a direct reconnection of the nerve immediately after injury, performed by an epineurial repair to suture the epineuria of the separated nerve endings. In a situation with a small intraneural connective tissue component, the best results occur when the nerves contain either purely sensory or purely motor components [2, 3]. Neurolysis is performed on intraneural and extraneural scar tissue to release degenerative nerve fibers with the hope of improving functional recovery [4]. In severely damaged nerves, a nerve graft would be necessary to achieve nerve continuity without tension [5].

Angiogenesis is a biological process in which new vessels are formed from old capillaries by sprouting or separating via several steps, including endothelial cell migration and proliferation [6]. Nerves and vessels constitute a complicated branching network within many tissues in the body, and they are closely correlated with each other in terms of anatomy and function [7]. Regarding vascular anatomy, specifically, the peripheral nervous system is a highly important participant in the angiogenesis process and so too are complete endothelial cells [8]. Increased rates of axonal regeneration in the vicinity of larger blood vessels and changes in capillary number and permeability are dependent upon successful axonal regeneration; this underscores the important interactions between axons and blood vessels [9]. In addition, the aspect of neoangiogenesis and neovasculogenesis by arteovenous loops and intellingent materials, both in experimental and clinical translation, has also been fully investigated [10–12].

Increased vascularization promotes axon regeneration capacity in an acellular nerve conduit [13]. In a nerve graft and conduit study, a neo-vascularization front preceded axonal regeneration and Schwann cells and axons extended together, never exceeding the area of vascularization; they appeared most numerous in well vascularized areas containing longitudinally oriented vessels [14]. In our previous study, we found that angiogenesis played a crucial role in promoting axon regeneration [15, 16]. Hence, either endogenous expression or exogenous supplement of angiogenesis was crucial for nerve regeneration [6–9, 13–20]. Therefore, wrapping injured nerve with the rotational muscle flap to increase angiogenesis may promote axon regeneration and be considered a valuable treatment strategy.

Based on the assumption of increased angiogenesis by the muscle flap wrapping the injured nerve to promote the nerve regeneration, we retrospectively analyzed our clinical data of severe radial nerve injury treated with neurolysis and rotational muscle flap. We also mimicked the clinical situation by nerve crush followed by rotation muscle flap in animals to assess the outcome of the expression of associated angiogenesis factors related to neurological outcomes.

## Materials and methods

### The patient population

From 2010 to 2017, there were 32 case diagnosed as the radial nerve injury obtained from the data bank. Among them, 4 cases of radial nerve paralysis with progressive improvement within 3 months and 3 cases of radial nerve rupture undergoing the sural nerve graft or nerve conduit repair were excluded from this study. Finally there were 25 cases of radial nerve injury without improvement over more than 6 months, and these were included in the clinical review. The patients included one case of osteomyelitis, 12 cases with inappropriate implantation, and 9 cases with chronic denervation. All patients underwent neurolysis and rotational muscle flap to wrap the injured nerve. The patients received periodic follow-up to assess muscle power and sensory function recovery, and they also underwent EMG/NCV testing. The outcomes of functional recovery were evaluated by Sakellarides’ scale for the sensory function and British medical research council scale for the muscle power [21, 22]. The reviews of medical charts and imaging were approved by the Taichung Veterans General Hospital Institute Review Board. The agreements of illustration of photography in the manuscript were also approved by the patients.

### Animal model

Sprague-Dawley rats weighing 250-300g were anesthetized with 4% isoflurane for induction and were kept under maintenance dose (1%-2%). The left sciatic nerve was exposed under a microscope using the gluteal muscle splitting method. The left sciatic nerve was crushed at the point of 10 mm from the obturator by a vessel clamps for 20 minutes [23]. These animals were randomly assigned to various treatment groups, including nerve crush alone (Crush), crush with rotational muscle flap (Crush+MF), and crush with rotational muscle flap and VEGF inhibitors (Avastin treatment) (Crush+MF+Avastin). The intramuscular injection over the rotational muscle flap consisted of 1.25 mg bevacizumab (Avastin) (Roche, Basel, Switzerland) according to the recommended dose for the treatment of the neo-vascular macular degeneration [24]. During the immediate post-procedural period, the animals were monitored closely in the recovery cage until they could maintain sternal recumbency. To minimize post-operative pain, the animals received an intramuscular injection of ketoprofen 5 mg/kg q12 hours for one day.

All animals underwent rehabilitation therapy on a metal mesh every week. Food and water were provided ad libitum before and after the experiments. The animals were kept in a temperature-controlled environment at 20 °C, and they were exposed to alternating light and dark cycles of 12 h intervals. The animals received neurological assessments weekly after surgery until the end of the experiment, and then they were subjected for histological and electrophysiological assessments at one month post-operation. After the experiment, all animals were euthanized with CO2.

All care and animal procedures were consistent with the ARRIVE guidelines (Animal Research: Reporting In Vivo Experiments). The study was approved by the Institutional Review Board and animal care complied with the Guide for the Care and Use of Laboratory Animals.

### Analysis of motor function of sciatic nerve-injured rats

Evaluation of sciatic nerve function was performed weekly for four weeks after the surgery [23]. The SFI was calculated based on the following equation: SFI = 38.3(EPL-NPL/ NPL) + 109.5(ETS-NTS/NTS) + 13.3(EIT-NIT/NIT) − 8.8. The SFI ratio with the SFI value of 0 was defined as normal function, and the SFI value of −100 was defined as complete injury.

### Nociceptive behavior

For behavior measurements, bilateral hind paws of all animals were examined by researchers blinded to management. Mechanical allodynia was assessed using von Frey hair (Touch-Test Sensory Evaluator) (North Coast Medical, Gilroy, CA, USA), as previously described by our group [25]. Briefly, a series of different grams von Frey hair was applied to the bilateral hind paw five times at 5 seconds intervals or at the moment that the hind paw was placed appropriately on the platform. The withdrawal threshold depended on the value (gram) of the hair that caused the hind paw to withdraw either four or five times out of the five applications. Thermal hyperalgesia was evaluated by hot-plate test (Technical & Scientific Equipment GmbH, Thuringia, Germany) according to our pervious procedure [25, 26]. The time of paw withdrawal latency was recorded during the course of the rat touching a 52–54 °C hotplate to the withdrawal of the paw. A protective proviso using a maximal cut-off of twenty seconds was maintained to prevent paw tissue injury.

### CatWalk gait analysis

CatWalk gait analysis (Noldus, Wageningen, Netherlands) was previously described by our group [25, 27]. Briefly, the CatWalk XT system comes with a high-speed digital camera with a sample rate of 100 frames per second. The brightness of a pixel depends on the amount of light received from such an area by the camera. The illuminated footprint enables intensity difference to be detected between animals’ paws. The intensity varies from 0 to 225, and they are represented by different colors. Quantitative analysis of the data from the CatWalk XT included the following parameters: step sequence distribution, regularity index, print area, duration of swing and stance phases, and footprint intensity.

### Electrophysiological assessment

The compound muscle action potential (CMAP) amplitudes and conduction latencies were recorded in the gastrocnemius muscle with an active monopolar needle electrode 10 mm below the tibia tubercle and with a reference needle 20 mm from the active electrode. The stimulation intensity and filtration ranges were 5 mA and 20–2000 Hz, respectively. A similar procedure was conducted on the other side as a control. The CMAP and conduction latency data were converted to the ratio of the injured side divided by the normal side to adjust for the effect of anesthesia [23].

### Enzyme-linked immunosorbent assays (ELISA)

The parts of nerves wrapped by muscle were obtained and subjected to homogenization in buffer containing 1% Triton X-100, 50 mM Tris-HCl, 150 mM NaCl, and 1% protease inhibitor cocktail (Cal-biochem) (Merck, Darmstadt, Germany). The homogenates and cultured supernatants were loaded onto 96-well plates at 4°C overnight. After washing with 0.1% Tween-20/PBS and blocking, the wells were incubated with indicated primary antibodies against von Willebrand (Santa Cruz Biotechnology, Dallas, TX, USA), isolectin B4 (Vector Laboratories, Burlingame, CA, USA), VEGF (Santa Cruz Biotechnology, Dallas, TX, USA) followed by biotin-conjugated secondary anti-body and streptavidin-HRP. Finally, the color was developed by the addition of TMB, and the optical density was measured using a microplate reader at wavelength 450 nm [28].

### Western blot analysis

The middle part of the gastrocnemius muscle and the distal end of crush nerves were harvested and proteins were extracted. Proteins (50 μg) were resolved by SDS-polyacrylamide gel electrophoresis and transferred to blotting membranes. After blocking with nonfat milk, the membranes were incubated with antibodies: S-100 (1:1000 dilution, Merck Millipore, Burlington, MA, USA), neurofilament (1:1000 dilution, Merck Millipore, Burlington, MA, USA), CD 68 (1:1000 dilution, Bio-rad, Hercules, CA, USA), von Willebrand factor (1:200 dilution, Santa Cruz Biotechnology, Dallas, TX, USA), Isolectin B4 (1:200 dilution, Vector Laboratories, Burlingame, CA, USA), desmin (1:1000 dilution, Abcam, Cambridge, MA,USA), acetylcholine receptor (1:1000 dilution, Merckmillipore, Burlington, MA, USA), GAPDH (1: 2000 dilution, Santa Cruz Biotechnology, Dallas, TX, USA) overnight at 4°C. The membranes were incubated with horseradish peroxidase-conjugated secondary antibody and developed using ECL western blotting reagents. The intensity of protein bands was determined by a computer image analysis system (IS1000) (Alpha Innotech Corporation, San Leandro, CA, USA) [29].

### Immunohistochemistry

The middle part of gastrocnemius muscle and distal end of crush nerve were harvested and then cryosectioned into 8-μm and mounted on Superfrost/Plus slides (Menzel-Glaser, Braunschweig, Germany). The tissue slices were subjected to immunohistochemistry with antibodies against von Willebrand factor (1:200 dilution, Santa Cruz Biotechnology, Dallas, TX, USA), isolectin B4 (1:200 dilution, Vector Laboratories, Burlingame, CA, USA), CD 68 (1:200 dilution, Bio-rad, Hercules, CA, USA), neurofilament (1:200 dilution, Merck Millipore, Burlington, MA, USA), S-100 (1:200 dilution, Merck Millipore, Burlington, MA, USA), desmin (1:200 dilution, Abcam, Cambridge, MA, USA), and acetylcholine receptor (1:200 dilution, Merck Millipore, Burlington, MA, USA), for detection of nerve and muscle regeneration/degeneration. The immunoreactive signals were observed using AF 488 donkey anti–mouse IgG and AF594 donkey anti-rabbit (1:200 dilution, Invitrogen, Carlsbad, CA, USA) under a confocal microscope [29].

### DiI and Fluorescein isothiocyanate–dextran perfusion for neovascularization quantitation

Four weeks after the operation, three animals were perfused using 1,1′-dioctadecyl-3,3,3′,3′-tetramethylindocarbocyanine perchlorate (DiI) fluorescent dye (Sigma-Aldrich, St. Louis, MO, USA) (20μl) in 200μl PBS from tail veins following a previously-described protocol and these animals were subjected to transcardial perfusion with 4% paraformaldehyde in 0.1 M phosphate buffer (pH 7.4) 24 hours after DiI injection [30]. The other 3 animals received transcardial perfusion of 125 μg fluorescein isothiocyanate–dextran (Sigma-Aldrich, St. Louis, MO, USA) in 500ml normal saline for 30 minutes [31]. Following euthanasia through intraperitoneal pentobarbital sodium injection, the parts of nerve wrapped by muscle were subjected to analysis. The quantitation method has been published by our group [29].

### Histology examination

After behavioral and electrophysiological examination, six rats in each group were subjected to transcardial perfusion with 4% paraformaldehyde in 0.1 M phosphate buffer (pH 7.4). Left sciatic nerves and adjacent muscles were harvested from the animals after electrophysiological assessment, and the nerve tissue was fixed on a plastic plate using stay suturing to keep the nerve straight. The nerve and muscle were embedded, cut longitudinally into sections 8-μm sections and examined with hematoxylin-eosin (H&E) and immunohistochemical staining [28].

### Statistical analysis

Data were presented as the mean ±standard error (SE). The statistical significance of differences between groups was determined by one–way analysis of variance (ANOVA) followed by Dunnett’s test. For SFI and Catwalk analysis, and von-Frey test, the results were analyzed by repeated-measurement of ANOVA followed by Bonferroni’s multiple comparison method. A *p* value less than 0.05 was considered significant.

## Results

### The clinical outcome of severe radial nerve treated by neurolysis and muscle flap rotation

**Fig 1:**
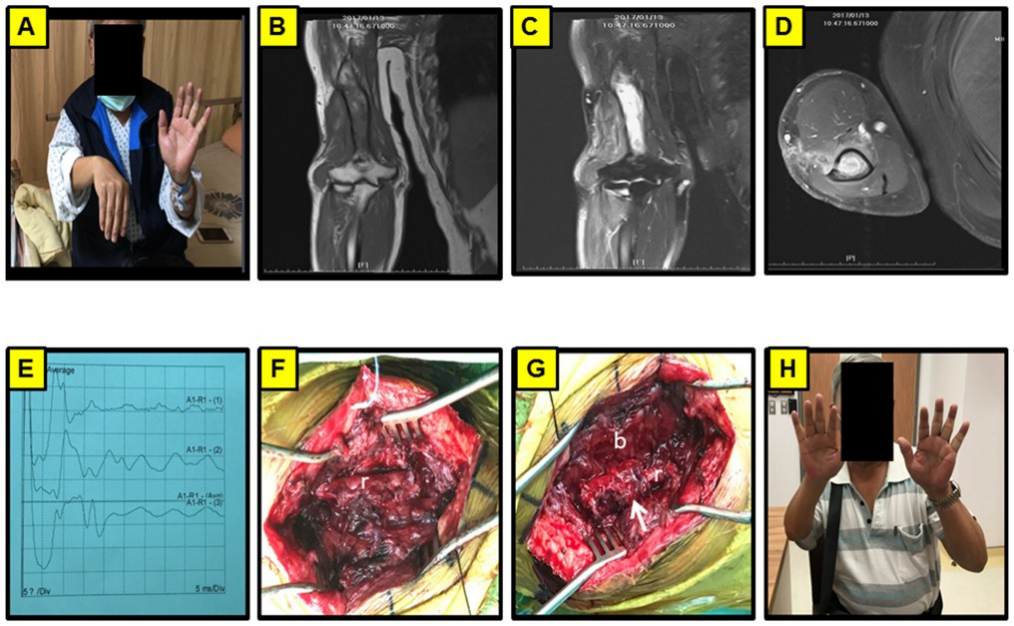
Representation of a right humerus fracture after ORIF complicated with osteomyelitis. (A) 68-year-old male presented with right wrist drop for 6 months after humerus fracture after operation. (B) MRI of right upper limb in T1W1 in coronal view. (C) MRI of right upper limb in T1W1 with contrast administration in coronal view. (D) MRI of right upper limb in T1W1 with contrast administration in axial view. (E) Intraoperative recording of CMAP stimulated from the distal to proximal end of the nerve. (F) The photography showed intact nerve but with severe fibrotic change. (G) Rotation of the muscle flap to wrap the injured region. (H) The patient showed favorable neurological outcome with restoration of wrist function 2 months after the operation. b: biceps muscle; r: radial nerve.

**Fig 2:**
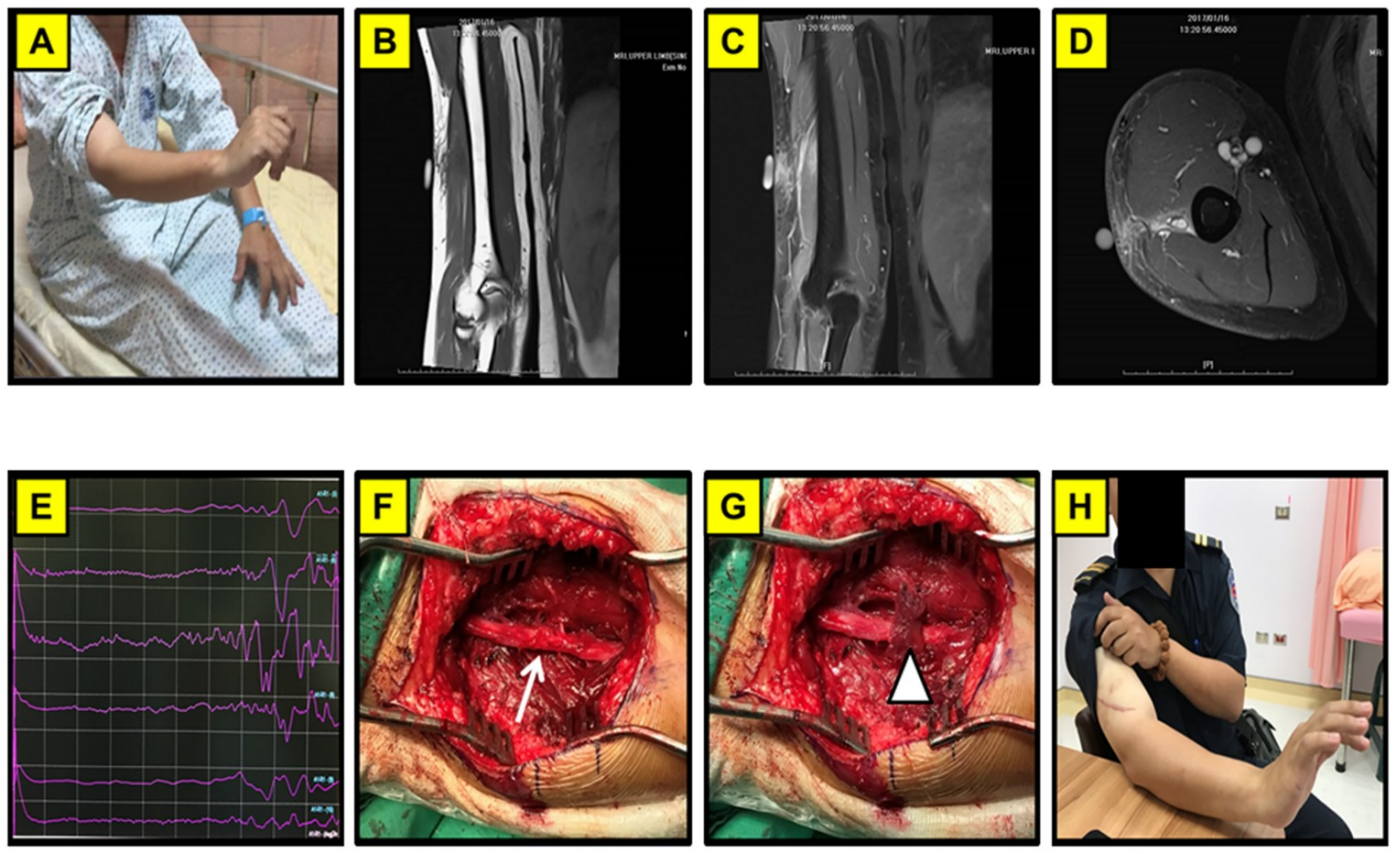
Representative right radial nerve injury by a crush injury with chronic denervation. (A) A 38-year-old male suffered a crush injury with the paralysis of finger dorsal flexion for 6 months. (B) MRI of right upper limb in T1W1 in coronal axial view. (C) MRI of right upper limb in T1W1 with contrast administration in coronal view. (D) MRI of right upper limb in T1W1 with contrast administration in axial view. (E) Intraoperative recording of CMAP in brachioradials and dorsal digitorum of radialis. (F) Intraoperative photography showing nerve crushed injury. (G) The injured nerve wrapped by the biceps muscle. (H) The patient showed the improved neurological outcome with restoration of wrist function 2 months after the operation. Arrow: radial nerve in the crush site; Arrow head: muscle flap rotation.

**Fig 3:**
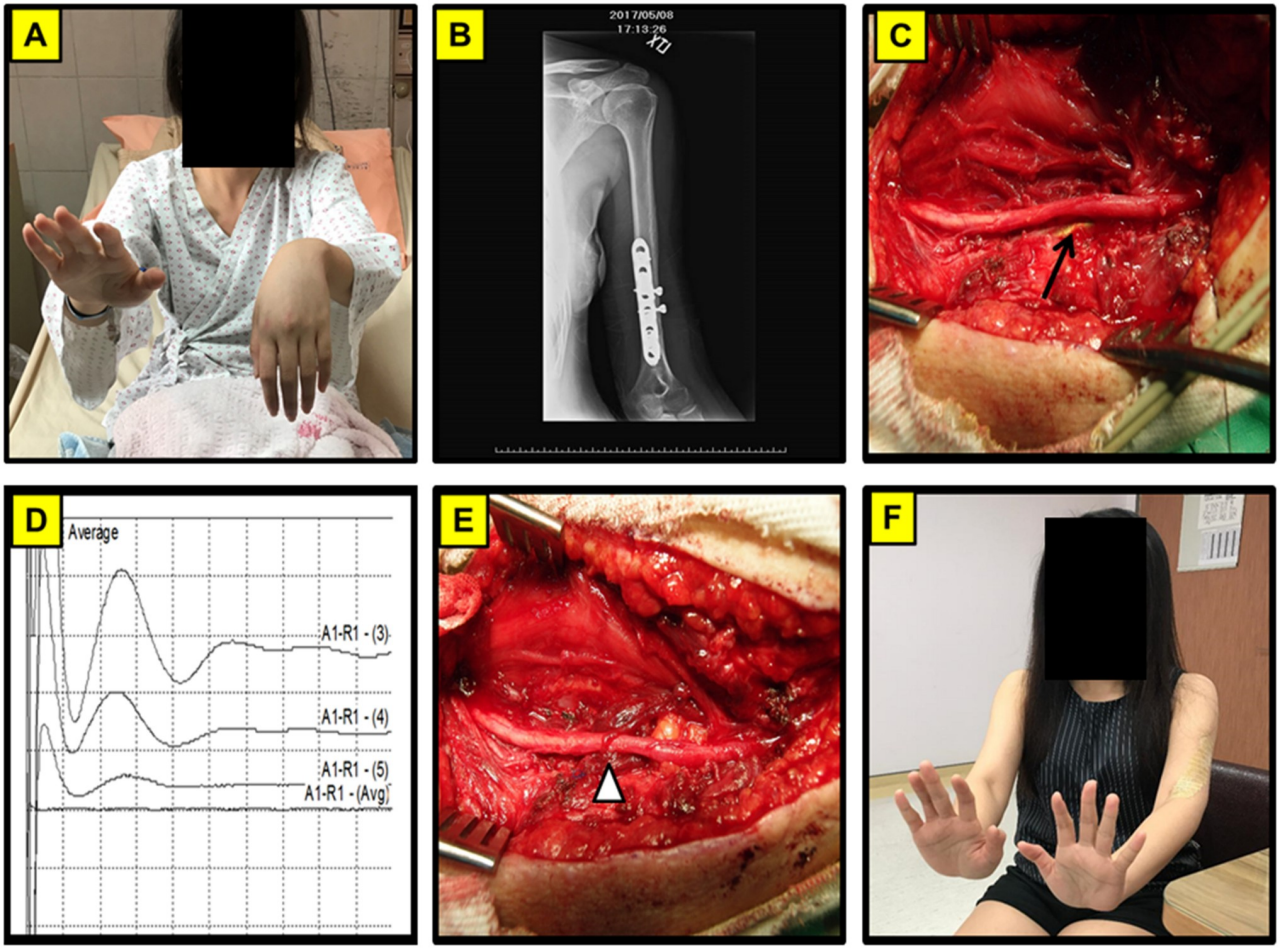
Representative left humerus fracture after ORIF complicated with nerve entrapped by the implant. (A) A 27-year-old female presented with left wrist drop for 6 months after a humerus fracture s/p operation repair of the fracture. (B) Plate and screws fixation in left humerus on X- ray film. (C) The photography showed the left radial nerve entrapped by the implant. (D) Intraoperative recording of CMAP stimulated from the distal to proximal end of nerve. (E) Rotation of a muscle flap to wrap the injured region and separation of the radial nerve from the implants. (F) The patient showed favorable neurological outcome with restoration of wrist function 3 months after operation. Arrow: implant; Arrow head: Muscles flap to wrap the radial nerve and separate it from the implant.

Twenty-five cases of severe radial nerve injury were evaluated in this study, and they consisted of one case of humerus fracture s/p ORIF complicated by osteomyelitis (Fig 1A–1G), 15 cases with impingement of nerve by the implants (Fig 2A–2H), and 9 cases of chronic denervation without improvement longer than 6 months (Fig 3A–3F). The mean age of the patients was 45 years (range 21–72 years). The ratio of female to male was 20 to 5. The mean duration of nerve palsy to treatment was 8 months (6–12). The onset of improvement of muscle power ranged from 1 to 6 months (mean 2 months). The details of motor and sensory function outcome as well as the name of rotational muscle flaps were depicted in Table 1. Following surgery, twenty-three of 25 (92%) cases gained muscle power to Grade 5, and 2 cases reached the muscle power of Grade 3. In Sakellarides scale analysis in the sensory function, 21 of 25 (84%) cases reach the scale of 5, 2 (8%) cases in the scale 4, and 2 (8%) cases in the scale 3. In the subsequent animal study, we mimicked the patients’ operative procedure by conducting nerve crush injury wrapped by rotational muscle flap to assess the power of angiogenesis involved in nerve regeneration.

**Table 1:** Outcome of motor /sensory function after muscle flap rotation.

	Pre op MRC	Post OP MRC	Pre-op Sakellarides scale	Post op Sakellarides scale	Muscle Flap
#1	0	5	3	5	Biceps
#2	0	5	2	5	Biceps
#3	0	5	2	5	Biceps
#4	0	3	2	3	Biceps
#5	0	5	3	4	Biceps
# 6	0	5	3	5	Biceps
# 7	0	3	2	3	Biceps
# 8	0	5	3	5	Biceps
# 9	0	5	2	5	Biceps
# 10	0	5	3	5	Biceps
# 11	0	5	2	5	Biceps
# 12	0	5	3	5	Biceps
# 13	0	5	2	5	Biceps
# 14	0	5	2	5	Biceps
# 15	0	5	3	5	Biceps
# 16	0	5	3	4	Biceps
# 17	0	5	2	5	Biceps
# 18	1	5	3	5	Biceps
# 19	0	5	2	5	Biceps
# 20	0	5	2	5	Biceps
# 21	0	5	2	5	Biceps
# 22	0	5	3	5	Biceps
# 23	1	5	2	5	Biceps
# 24	0	5	3	5	Biceps
# 25	1	5	3	5	Biceps

MRC: British medical research council scale for the muscle power

### Improvement of neurological outcome after muscle flap rotation and abolished by Avastin injection

**Fig 4:**
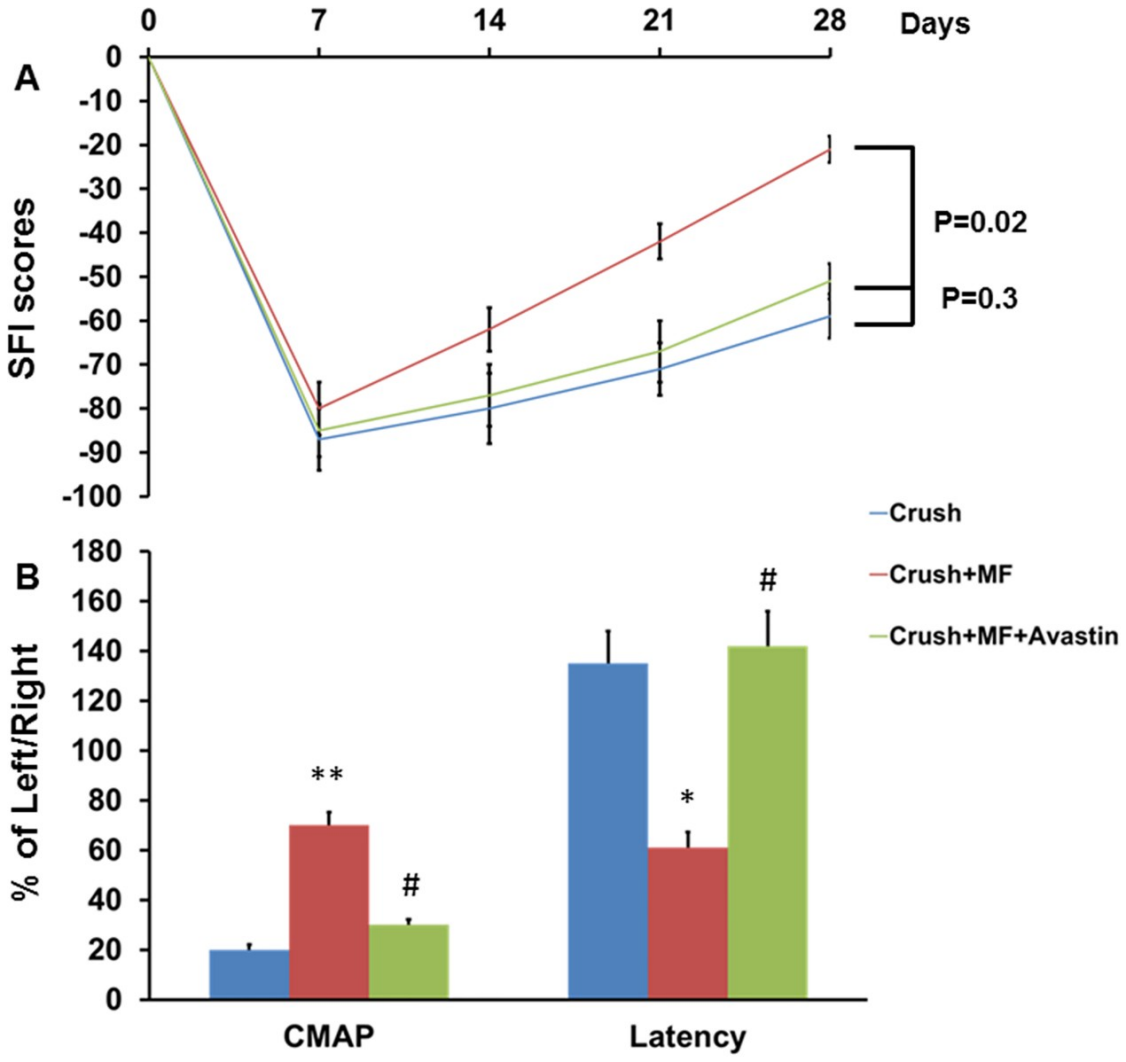
Representative SFI and electrophysiology results. There were three groups of animals subjected to SFI analysis weekly and then electrophysiology 4 weeks after injury. (A) The data of SFI related to different time points in the three different groups (B) CMAP and conduction latency presented as the ratio of left side/right side. Crush, Crush+MF, Crush+MF+Avastin: see text. *p<0.05, **p<0.01 indicated the Crush+MF related to Crush, # p<0.05 indicated Crush+MF+Avastin related to Crush+MF.

The Crush+ MF group showed significant improvement of SFI compared to the Crush and Crush+MF+Avastin groups (p = 0.02). However, there were no significant difference between the Crush and Crush+MF+Avastin groups (Fig 4A). On electrophysiology study, the CMAP in the Crush+MF group was 70±5.2%, significant improvement compared to the Crush (20±2.1%) (p<0.001) and Crush+ MF+ Avastin (30±2.3%) groups (p<0.001). The conduction latency in the Crush+MF group was 61±6.3%, which showed a significant improvement compared to the Crush (135±12.9%) (p<0.01) and Crush+MF+ Avastin (142±3.8%) groups (p<0.05) (Fig 4B). In the CatWalk gait analysis, increased printed area, Max contact maximum intensity, and stand, as well as decreased swing were significantly higher in the Crush+MF group than in the Crush and Crush+MF+Avastin groups (Table 2). In the thermal and mechanical withdrawal experiments, there was no significant differences among these groups (Table 3).

**Table 2:** Outcome of catwalk gait analysis in various treatment groups related to various time frames and catwalk parameters.

		0	3	7	14	21	28	P value
Printed area (% of Lt/Rt)	Crush	98±0.7	45±3.8	47±5.1	51±4.2	59±5.4	63±5.9	P<0.01
	Crush+ MF	99±1.1	47±4.1	50±3.9	66±4.7	77±5.1	89±4.8	
	Crush+ MF+ Avastin	97±1.1	44±4.2	47±3.3	60±5.2	65±4.7	67±5.1	
Max Contact Maximum Intensity(% of Lt/Rt)	Crush	95±0.9	70±5.5	71±6.3	75±5.9	79±7.3	82±6.5	P<0.01
	Crush+ MF	96±1.1	71±5.8	77±8.7	83±6.2	89±7.3	98±4.9	
	Crush+ MF+ Avastin	97±0.8	69±4.8	72±6.1	74±8.1	78±5.5	84±4.7	
Stand(% of Lt/Rt)	Crush	101±1.2	60±3.8	62±4.4	69±7.2	73±6.5	79±4.9	P<0.05
	Crush+ MF	98±0.9	62±4.2	67±3.9	78±5.2	85±6.7	94±7.3	
	Crush+ MF+ Avastin	102±0.5	63±4.2	64±5.1	68±5.7	74±4.9	81±5.2	
Swing (% of Lt/Rt)	Crush	102±0.8	201±17.8	198±15.4	170±11.2.	160±9.8	150±10.2	P<0.05
	Crush+ MF	98±0.6	199±16.5	190±13.8	140±9.7	121±9.2	102±8.4	
	Crush+ MF+ Avastin	101±1.1	211±15.8	197±12.7	167±8.8	158±7.9	149±8.5	

The data was presented as the mean±standard error

Crush, Crush+MF, Crush+MF+Avastin: see text

**Table 3:** Outcome of von Frey and thermal plate in different treatment groups related to various time frames.

		0	3	7	14	21	28	P value
Von Frey (gm)	Crush	72±2.1	35±4.1	28±2.9	38±2.5	45±3.2	62±2.8	0.55
	Crush+ MF	71±1.7	33±3.1	27±3.4	41±2,1	46±2.8	63±2,5	
	Crush+ MF+ Avastin	73±1.1	36±3.8	29±4.2	39±3.2	44±2.1	64±3.1	
Thermal plate (seconds)	Crush	15±1.1	25±2.1	30±2.8	24±1.9	21±2.8	18±1.3	0.75
	Crush+ MF	17±0.9	26±1.9	31±2.1	27±1.7	24±2.2	19±1.5	
	Crush+ MF+ Avastin	16±0.9	27±1.9	32±2.1	28±1.7	23±2.4	20±1.5	

The data was presented as the mean±standard error

Crush, Crush+MF, Crush+MF+Avastin: see text

### The increased angiogenesis and regeneration of crushed nerve by muscle flap rotation

**Fig 5:**
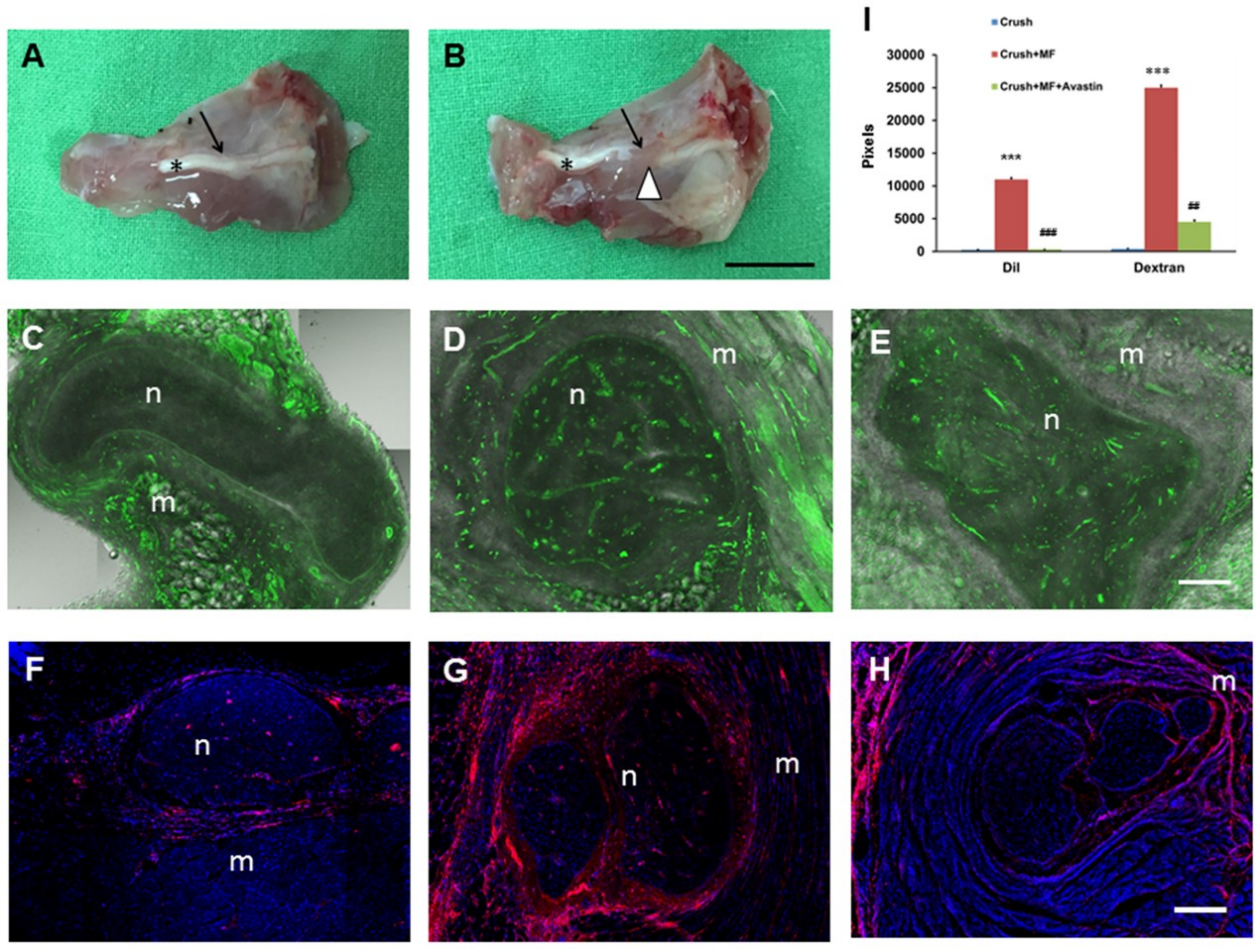
Representative muscle flap rotation and potential of angiogenesis results. (A) A representative nerve crush. (B) Nerve crush wrapped by the muscle. (C) Demonstration of FITC dextran infusion in the Crush group. (D) Demonstration of FITC dextran infusion in the Crush+MF group. (E) Demonstration of FITC dextran infusion in the Crush+MF+Avastin group. (F) Demonstration of DiI infusion in the Crush group. (G) Demonstration of DiI infusion in the Crush+MF group. (H) Demonstration of DiI infusion in the Crush+MF+Avastin group. (I) Quantitative analysis of FITC dextran and DiI infusion in various treatment groups. Crush, Crush+MF, Crush+MF+Avastin: see text. *p<0.05, **p<0.01 indicated the Crush+MF related to Crush, # p<0.05 indicated Crush+MF+Avastin related to Crush+MF. Black arrow bar length = 1cm; White arrow bar length = 100 μm; Black arrow: crush site; white arrow head: muscle flap. n: center of nerve; m:muscle.

**Fig 6:**
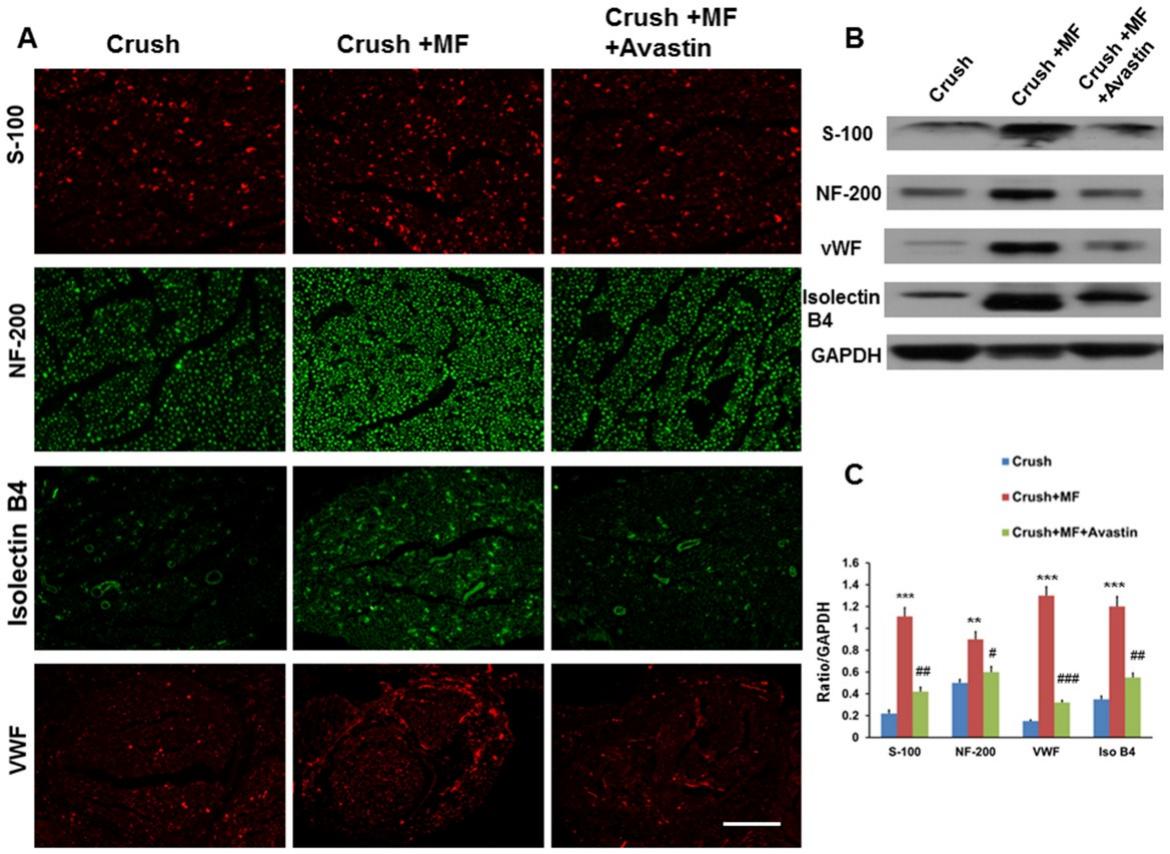
Increased nerve regeneration and angiogenesis by the muscle flap rotation. (A) The representative of crushed nerve in the expression of S-100, NF, isolectin B4, and vWF in various treatment groups. (B) A representative of western blot showing expression of S-100, NF, isolectin B4, and vWF in the various treatment groups. (C) Quantitative analysis of S-100, NF, isolectin B4, and vWF in the various treatment groups. Crush, Crush+MF, Crush+MF+Avastin: see text. *p<0.05, **p<0.01 indicated the Crush+MF related to Crush, # p<0.05 indicated Crush+MF+Avastin related to Crush+MF. Arrow bar length = 100 μm.

**Fig 7:**
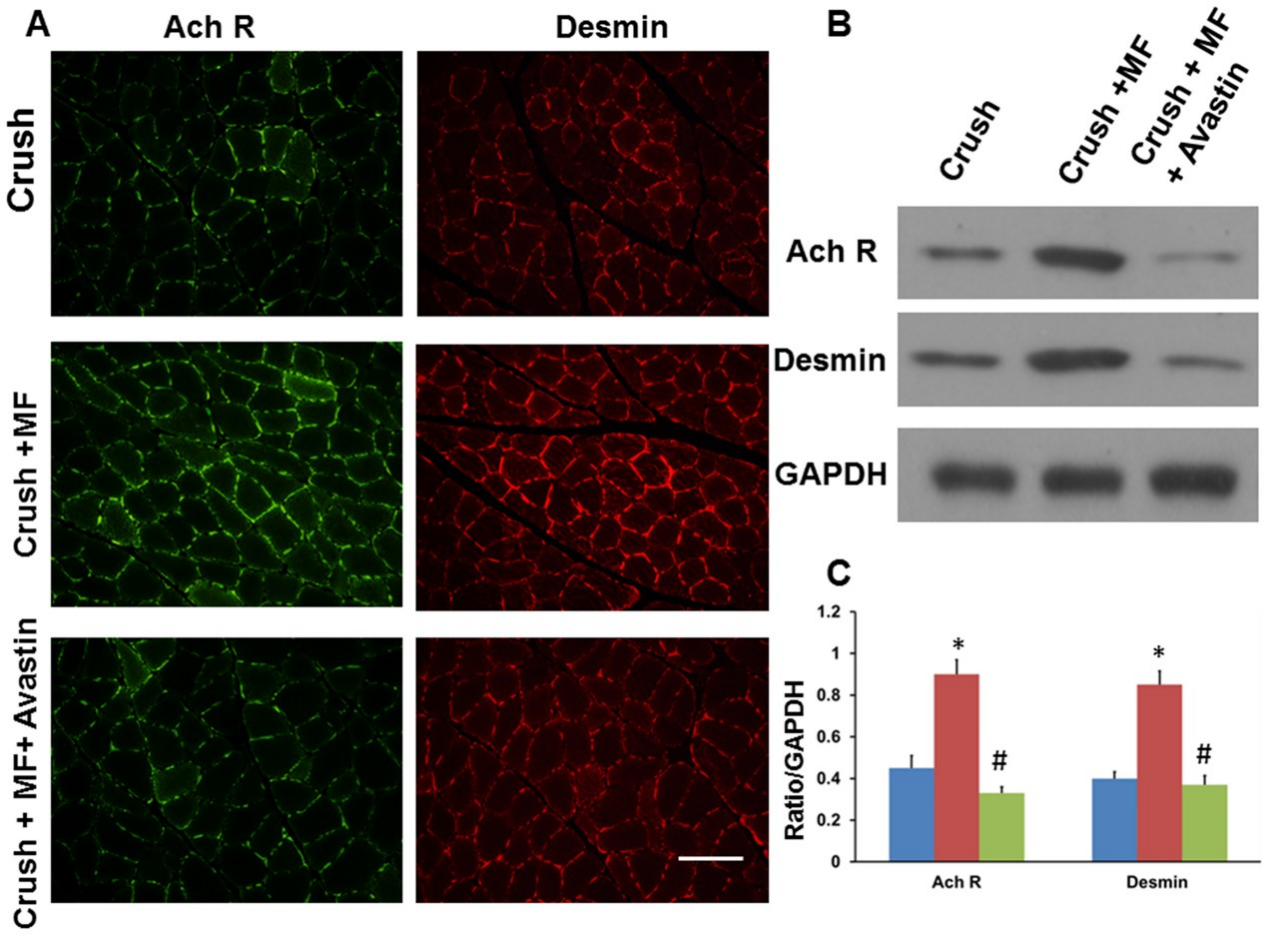
Restoration of morphology in denervated muscle in the crushed nerve wrapped by muscle flap rotation. (A) A representative example of a denervated muscle showing the expression of acetylcholine receptor and desmin in various treatment groups. (B) A representative example of western blot showing expression of acetylcholine receptor and desmin in the various treatment groups. (C) Quantitative analysis of acetylcholine receptor and desmin in the various treatment groups. Crush, Crush+MF, Crush+MF+Avastin: see text. *p<0.05, **p<0.01 indicated the Crush+MF related to Crush, # p<0.05 indicated Crush+MF+Avastin related to Crush+MF. Arrow bar length = 100 μm.

The architecture of muscle flap rotation related to the crushed nerve is shown in Fig 5A and 5B. FITC-dextran infusion showed appreciable green fluorescence distributed to the nerve in the Crush+MF group compared to the Crush group, and these effects were attenuated by Avastin injection (Crush+MF+Avastin) (Fig 5C–5E). The DiI dye injection also showed the same phenomenon as that of FITC-dextran infusion (Fig 5F–5I). These crushed nerves were allocated for determination of angiogenesis factors and nerve regeneration potential. Levels of von William factor (vWF) in Crush+MF showed an 8.6-fold increase over those of the Crush group (p<0.001). The effect was also attenuated by Avastin treatment, as seen in the Crush+MF+Avastin group (p<0.01). Isolectin B4 levels showed a 3.4- fold increase compared to the Crush group (p<0.001), and this was attenuated by Avastin treatment, as seen in the Crush+MF+Avastin group (p<0.01). There were significantly higher expression levels of S-100 and neurofilament in the Crush+MF group than in the Crush group (p<0.001, p<0.01, respectively). These expression levels were significantly reduced by Avastin injection in the Crush+MF+Avastin group (p<0.01, p<0.05) (Fig 6A–6C). The ELISA analysis also showed similar trends (Table 4). Furthermore, the restoration of denervated muscle morphology in the expression of acetylcholine and desmin were in line with the trend of increased nerve regeneration (Fig 7A–7C).

**Table 4:** ELISA analysis of angiogenic factors of nerve wrapped by the muscle flap.

	Crush	Crush+MF	Crush+MF+Avastin	P value
von Willebrand	0.35±0.04 OD	0.78±0.04 OD	0.45±0.05 OD	P<0.05
Ioslectin B4	0.45±0.06 OD	0.88±0.07 OD	0.51±0.07 OD	P<0.05
VEGF	0.31±0.02 OD	0.55±0.04 OD	0.32±0.04 OD	P<0.05

The data was presented as the mean±standard error

Crush, Crush+MF, Crush+MF+Avastin: see text

**Fig 8:**
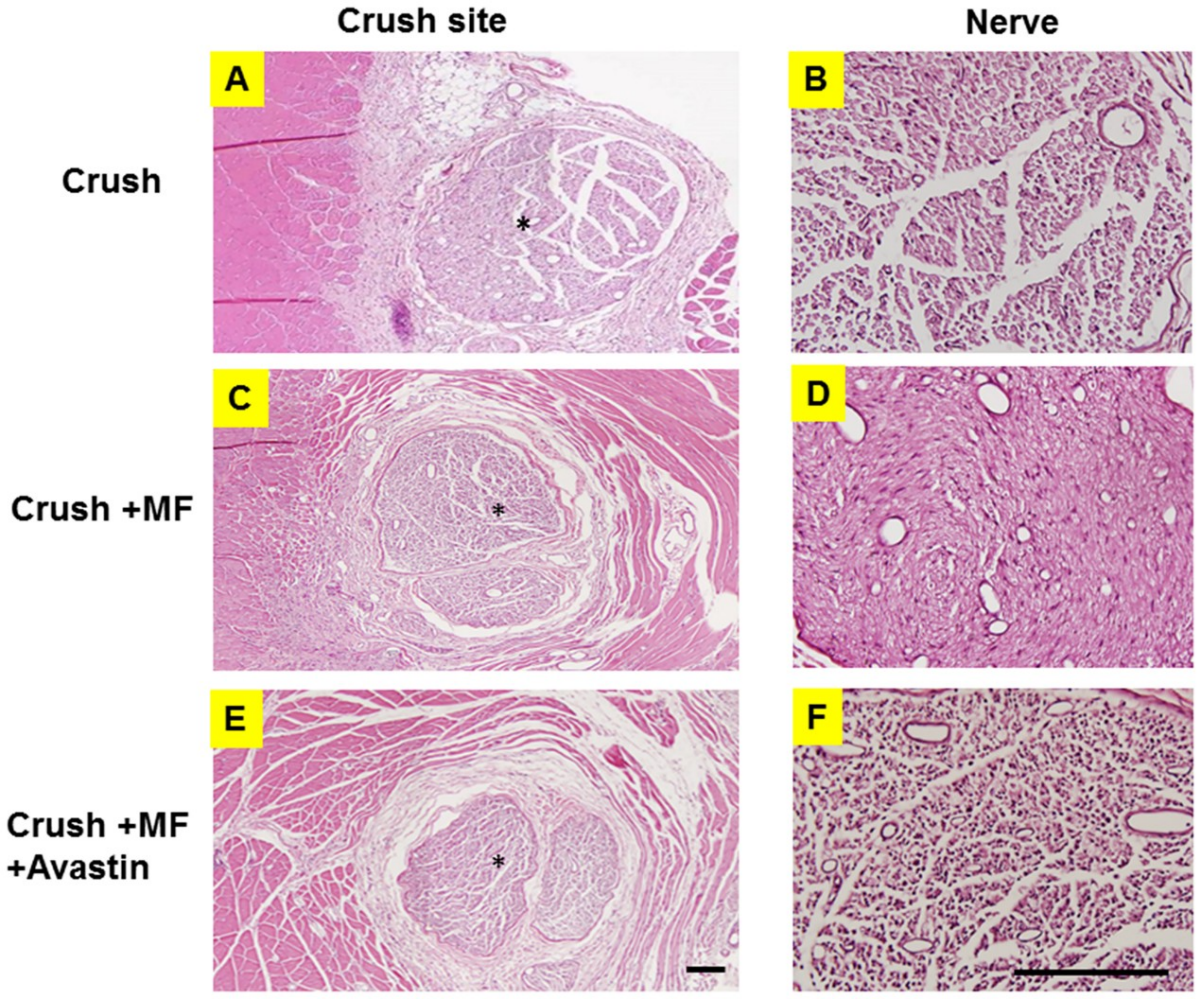
Histomorphology analysis of nerve related to muscle flap. (A) Illustration of crush neve related to muscle flap in the Crush group. (B) Loss of nerve intact structure in the Crush group. (C) Illustration of crushed neve related to muscle flap in the Crush group. (D) Well-organized nerve structure with increased vessel structure in the Crush+ MF group. (E) Illustration of crushed neve related to muscle flap in the Crush+ MF+ Avastin group. (F) Less-organized nerve structure in the Crush+ MF+ Avastin group. *: center of nerve. Arrow bar length = 100 μm.

In H&E analysis, the muscle flap showed integration of nerve related to the muscle flap. There were remarkably increased numbers of vessel structures among the intraneural structures in the Crush+ MF group compared to the Crush group, and the phenomena of increased numbers of vessel structures was attenuated by Avastin treatment, as seen in the Crush+ MF+ Avastin group (Fig 8A–8F). In the assessment of axon count number and morphology, there were significantly increased numbers of axon and greater proportions of large diameter axons in the Crush+ MF group than in the Crush and Crush+ MF+ Avastin groups (Table 5).

**Table 5:** Axon counts and diameter in various groups.

	Crush	Crush+ MF	Crush+ MF+ Avastin	P value
Number of axons /0.1mm^2^	67.5±4.1	91.7±8.4	71.4±5.9	<0.01
Ratio of axon diameter<2μm (%)	68±7	51±5	65±7	<0.05
Ratio of axon diameter>2μm (%)	32±4	49±4	35±4	<0.05

The data was presented as the mean±standard error

Crush, Crush+MF, Crush+MF+Avastin: see text

## Discussion

Increase angiogenesis in crushed nerve mediated by the rotation muscle flaps is a crucial factor in nerve regeneration, as shown in both clinical and animal studies. The integration of injured nerve to a rotational muscle flap showed the ingrowth of vessels from the muscle and led to increased expression of associated angiogenesis factors. The abolishment of the angiogenesis effect by intramuscular Avastin injection caused a reciprocal effect, and confirming this hypothesis. Furthermore, there were no adverse effects such as increased pain sensory threshold after muscle flap rotation. Therefore, the strategy of muscle flap rotation in the assistance of nerve regeneration could be a treatment option in nerve repair.

Endogenous angiogenesis is a biological process responded to injury in which new vessels form from old capillaries, and the integration of nerves and vessels constitute a complicated branching network within the injured nerve [6, 7]. Neurobehavior, gastrocnemius muscle mass, and morphometric indices confirmed faster recovery of regenerated axons with VEGF administration. On Immunohistochemical assessment, reactions to S-100 in the VEGF group were more positive than those in a silicone group [18]. Local application of VEGF promotes the invasion of Schwann cells and neovascularization, both of which are important for nerve regeneration [19]. There, both the endogenous and exogenous supplementation of angiogenesis factors are essential for nerve regeneration and this further confirm our hypothesis.

Avastin is an immunoglobulin G monoclonal antibody directed against VEGF, used for the treatment of cancer and aged-related macular degeneration [24, 32]. In this study, rotational muscle augmented the vascular structures over injured nerves and the associated angiogenesis factor levels. At the same dosage used in macular degeneration [24], intramuscular injection of Avastin attenuated the microvascular structures and angiogenesis factors in the crushed nerve. The up-and-down regulation of angiogenesis factors in the crushed nerve paralleled the increased and decreased nerve regeneration demonstrated in this study, and the results highlight the effects of angiogenesis contributing to nerve regeneration.

There remains a debate concerning the extent to which angiogenesis is beneficial or detrimental to nerve degeneration. Several lines of evidence suggest that pathological angiogenesis caused by the VEGF cascade in the inflammatory state is regulated by circulating leukocytes [33]. Angiogenic properties of macrophages and neutrophils are stimulated by chemokines and the impact of circulating neutrophils and macro-phages in angiogenesis may contribute to the development of neuropathic pain [34, 35]. In this study, the increased angiogenesis around nerves by the muscle flap rotation did not promote the recruitment of inflammatory cells (S1 Fig) involved in inflammatory responses. Furthermore, there was no increase in nociceptor sensation after angiogenesis by muscle flap rotation. Therefore, the rotational flap did not increase the expression of inflammatory responses in the injured nerve nor did angiogenesis contribute to the development of neuropathic pain in the current study.

## Conclusion

In our clinical review, the rotation of muscle flap after neurolysis led to an appreciable neurological improvement after surgery. The analogous preclinical study showed that muscle flap rotation augmented angiogenesis in the injured nerve. The increased microvascular structure was associated with nerve regeneration and favorable neurological outcome. Therefore, the combined neurolysis and rotation of muscle flap to wrap the injured nerve appears to be a reasonable adjuvant treatment option for nerve repair.

## Supporting information

S1 Checklist NC3Rs ARRIVE Guidelines Checklist. (PDF)

S1 Fig Depiction of inflammatory response in crushed nerve by the muscle flap rotation and Avastin injection. (A) The illustration of CD 68 distributed in crushed nerve subjected to various treatments. (B) A representative of western blot showing expression of CD 68 in the various treatment groups. (C) Quantitative analysis of CD 68 in the various treatment groups. Crush, Crush+MF, Crush+MF+Avastin: see text. Arrow bar length = 100 μm.
(TIF)
