# Supplementary material for: Increased angiogenesis by the rotational muscle flap is crucial for nerve regeneration
Source: PLoS One. 2019 Jun 10;14(6):e0217402. doi: 10.1371/journal.pone.0217402 (PMC6557495; doi:10.1371/journal.pone.0217402)
Supplement: S1 Checklist — (PDF) [file pone.0217402.s001.pdf]

ARRIVE

The ARRIVE Guidelines Checklist

	ITEM	RECOMMENDATION	Section/ Paragraph
Title	1	Inceased angiogenesis by the rotational muscle flap is crucial for nerve regeneration	Page 1
Abstract	2	Background:<br>The gold standard surgical treatment of nerve injury included the direct repair, nerve graft, and neurolysis. The process of angiogenesis during the nerve regeneration by rotational muscle flap either beneficial or detrimental was not determined yet.<br>Objective:<br>We assess the neurological outcome and angiogenesis of nerve injury followed by the rotational muscle flap.<br>Methods:<br>We retrospectively analyze the outcome of the patients with severe radial nerve injury by neurolysis and rotational muscle flap and also mimic the clinical situation by nerve crush followed by rotation muscle flap in animals to assess the associated angiogenesis factors expression.<br>Results:<br>Twenty three of 25 (92%) cases of severe radial nerve injury underwent the neurolysis assisted by the muscle flap rotation reached the pre-injury neurological outcome. In the animal study, either of FITC–dextran or DiI infusion showed the remarkable increased vascular structure in the crushed nerve integrated by muscle flap and abolished by Avastin injection. The rotational muscle flap significantly increased von-William factors and Isolectin B4 expression and attenuated by the Avastin injection. These increased angiogenesis factors expression paralleled the improvement of neurobehavior and electrophysiological study as well as the significant expression of nerve regeneration marker and restoration of denervated muscle morphology.<br>Conclusion:<br>Based on the clinical and animal data analysis, the muscle flap rotation provides a platform for angiogenesis in the acceleration of nerve regeneration. It implicated that the rotational flap rotation augmented the nerve regeneration process which may be beneficial for the nerve repair in clinical application.	Page 1-2
INTRODUCTION			
Background	3	The gold standard treatment of peripheral nerve injury is hopeful to return pre-injury function of the damaged nerve and, at minimum, to improve the quality of life of patients (1). Primary repair is a direct reconnection of the nerve immediately after injury. In an epineuria repair, the epineuriums of the separated nerve endings are sutured together using a microsuture. The best results occur when the nerves are either purely sensory or purely motor and when the intraneural connective tissue component is small (2, 3). Neurolysis is performed on intraneural and extraneural scar tissue to release degenerative nerve fibers	Page 3-5

		in the hope of improving functional recovery (4). Contaminated wounds, such as gunshot wounds and avulsions with severe tissue disruption, benefit from a second nerve repair (4). In severely damaged nerves, a nerve graft would be necessary and the defect should be closed without tension (5).<br><br>Angiogenesis is a biological process in which new vessels are formed from old capillaries by sprouting or separating via multiple steps, such as endothelial cell migration and proliferation (6). Nerves and vessels constitute a complicated branching network within many tissues in the body, and are closely correlated with each other in terms of anatomy and function (7). Regarding vascular anatomy, specifically, the peripheral nervous system is a highly important participant in the angiogenesis process, as are complete endothelial cells (8). Increased rates of axonal regeneration in the vicinity of larger blood vessels and changes in capillary number and permeability are dependent upon that successful axonal regeneration suggests an interaction between axons and blood vessels (9).<br><br>Increased vascularization promotes the axon regeneration capacity in a acellar neve conduit (10). In nerve graft or conduit study, a neo-vascularization front preceded axonal regeneration and Schwann cells and axons extended together, never exceeding the area of vascularization and appeared most numerous in well vascularized areas containing longitudinally orientated vessels (11). In our previous study, we also found that angiogenesis played a crucial role to promote the axon regeneration (12, 13) .Hence, either endogenous expression or exogenous supplement of angiogenesis was very crucial for nerve regeneration (6-17). Thus, increased angiogenesis in injury nerve wrapped by the rotational muscle flap to augment angiogenesis to promote axon regeneration may be considered to be a treatment strategy.	
Object	4	Based on the assumption of increased angiogenesis by the muscle flap to wrap the injured nerve to promote the nerve regeneration, we retrospectively analyze our clinical data of severe radial nerve injured treated with neurolysis and rotational muscle flap and also mimic the clinical situation by nerve crush followed by rotation muscle flap in animals to assess the outcome of the expression of associated angiogenesis factors related to neurological outcome.	Page 4-5
METHODS			
Ethic statement	5	All care and animal procedures were consistent with the ARRIVE guidelines (Animal Research: Reporting In Vivo Experiments). The study was approved by the Institutional Review Board and that the animal care complied with the Guide for the Care and Use of Laboratory Animals.Sprague-Dawley rats weighing 250-300 g were used in this study. Permission was obtained from Taichung Veterans General Hospital Institutional Animal Care and Use Committee (IACUC) (Permission No.La-1061478).	Page 5
Study deisgn	6	The left sciatic nerve was exposed under a microscope using the gluteal musc*le splitting method. The left sciatic nerve was crushed at the poi*nt of 10 mm from the obturator by a vessel clamps for 20 minutes (18). These animals were allowed to receive different treatment including nerve crush alone (Crush), crush with rotational muscle flap (Crush+MF), and crush with rotational muscle flap and VEGF inhibitors (Avastin treatment)	Page 5-6

		(Crush+MF+Avastin). The intramuscular injection over the rotational muscle flap was 1.25 mg bevacizumab according to recommended dose of treatment in the neo-vascular macular degeneration (19).	
Experimental Procedure	7	The left sciatic nerve was exposed under the microscope using the gluteal muscle splitting method. Based upon prior reports from our lab, a vessel clamp (B-3, pressure 1.5g/mm2, S&T Marketing Ltd, Neuhausen, Switzerland) was used 10 mm from the internal obturator canal for 20 minutes as the crush injury model. These animals were allowed to receive different treatment including nerve crush alone (Crush), crush with rotational muscle flap (Crush+MF), and crush with rotational muscle flap and VEGF inhibitors (Avastin treatment) (Crush+MF+Avastin). The intramuscular injection over the rotational muscle flap was 1.25 mg bevacizumab according to recommended dose of treatment in the neo-vascular macular degeneration (19).The wound was closely observed and evaluated every day and the stitches were removed 10 days after operation. The rehabilitation program was conducted on a metal mesh every week. The animal housing environment was kept in the appropriate condition with 2 animals in a single cage, in a temperature-controlled environment at 20 ℃ and alternating light and dark cycles with 12 hour intervals. After the experiment, all animals were euthanized with CO2. All animals of care and operation were according to the guideline of ARRIVE (Animal Research: Reporting In Vivo Experiments). The animals received neurological assessment (SFI, Catwalk gait analysis) pre-operative and weekly after operation till the end of experiment and then subjected for histomorphological assessment(H&E) and electrophysiology(CMAP, conduction latency) 4 weeks after operation (n=6 for each group)(total of 18 animals). At the end of experiment, nerve and muscle tissue of these animals were also used for immunohistochemistry staining (n=6), western blot analysis (n=6), ELISA (n=6), DiI and FITC-dextran (n=6) (total of 72 animals).	Page 5-6
Experimental Animals	8	Male Sprague-Dawley rats weighing 250-300 g were bought from BioLASCO Taiwan Co., Ltd and used in this study.	Page 5
Housing and husbandry	9	The animal housing environment was kept in the appropriate condition with 2 animals in a single cage, in a temperature-controlled environment at 20 ℃ and alternating light and dark cycles with 12 hour intervals.	Page 6
Sample size	10	These animals were allowed to receive different treatment including nerve crush alone (Crush), crush with rotational muscle flap (Crush+MF), and crush with rotational muscle flap and VEGF inhibitors (Avastin treatment) (Crush+MF+Avastin). The animals received neurological assessment (SFI, Catwalk gait analysis) pre-operative and weekly after operation till the end of experiment and then subjected for histomorphological assessment(H&E) and electrophysiology(CMAP, conduction latency) 4 weeks after operation (n=6 for each group)(total of 18 animals). At the end of experiment,	Page 5-6

	nerve and muscle tissue of these animals were also used for immunohistochemistry staining (n=6), western blot analysis (n=6), ELISA (n=6), DiI and FITC-dextran (n=6) (total of 72 animals).	
Allocating animals To experimental group	11 These animals were allowed to receive different treatment including nerve crush alone (Crush), crush with rotational muscle flap (Crush+MF), and crush with rotational muscle flap and VEGF inhibitors (Avastin treatment) (Crush+MF+Avastin).	Page 5-6
Experimental Outcome	12 The neurological outcome, electrophysiology, nerve architecture, muscle morphology were obtained for analysis.	Page 12-15
Statistical Method	13 Data was presented as mean ±standard error (SE). The statistical significance of differences between groups was determined by one–way analysis of variance (ANOVA) followed by Dunnett's test. For SFI and Catwalk analysis, Von-Frey test, the results were analyzed by repeated-measurement of ANOVA followed by Bonferroni's multiple comparison method. A p value less than 0.05 was considered to be significant.	Page 12 Line 5-11
RESULTS		
Baseline Data	14 In the animal study, either of FITC–dextran or DiI infusion showed the remarkable increased vascular structure in the crushed nerve integrated by muscle flap and abolished by Avastin injection. The rotational muscle flap significantly increased von-William factors and Isolectin B4 expression and attenuated by the Avastin injection. These increased angiogenesis factors expression paralleled the improvement of neurobehavior and electrophysiological study as well as the significant expression of nerve regeneration marker and restoration of denervated muscle morphology.	Page 12-15
Number analyzed	15 The animals received neurological assessment (SFI, Catwalk gait analysis) pre-operative and weekly after operation till the end of experiment and then subjected for histomorphological assessment(H&E) and electrophysiology(CMAP, conduction latency) 4 weeks after operation (n=6 for each group)(total of 18 animals). At the end of experiment, nerve and muscle tissue of these animals were also used for immunohistochemistry staining (n=6), western blot analysis (n=6), ELISA (n=6), DiI and FITC-dextran (n=6) (total of 72 animals).	Page 12-15
Outcome And estimation	16 . Improvement of neurological outcome after muscle flap rotation and abolished by Avastin injection<br>The Crush+ MF group showed the significant improvement of SFI as compared to Crush or Crush+ MF+ Avastin group (p=0.02). But there were no significant difference between Crush and Crush+ MF+ Avastin group (Figure 4 A). In electrophysiology study, the CMAP in Crush+MF group were 70±5.2 %, which showed the significant improvement as compared to Crush (20±2.1%)(p<0.001) and Crush+ MF+ Avastin (30±2.3%) (p<0.001). The conduction latency in	Page 12-15

		Crush+MF group were 61±6.3 %, which showed the significant improvement as compared to crush (135±12.9%) (p<0.01) and Crush+MF+ Avastin (142±3.8%) (p<0.05) (Figure 4 B). In the CatWalk analysis, the increased printed area, Max contact maximum intensity, and stand as well as decreased swing were significantly high in Crush+MF either as compared to Crush or Crush+MF+Avastin (Table I). In the thermal and mechanical withdraw; there was no significant difference among these groups (Table II)<br><br>The increased angiogenesis and regeneration of crushed nerve by the muscle flap rotation<br>The architecture of muscle flap rotation related to the crushed nerve was showed in Figure (5A). The DiI dye infusion showed the remarkable DiI dye distributed to nerve in Crush+MF group as compared to Crush and these effects were attenuated by Avastin injection (Crush+MF+Avastin). The FITC-dextran injection also showed the same phenomenon as those of DiI infusion (Figure 5B-D). These crushed nerves were allocated for the determination of angiogenesis factors and nerve regeneration potential. The angiogenesis factors of Von William factors (vWF) in Crush+MF showed 8.6 fold increase as compared to crush group (p<0.001). The effect was also attenuated by Avastin treatment (Crush+MF+Avastin group) (p<0.01). The Isolectin B4 also showed 3.4 fold increased as compared to the crush group (p<0.001) and attenuated by Avastin treatment (Crush+MF+Avastin) (p<0.01). There were significant expressions of S-100 and neurofilament in the Crush+MF group as compared to crus group (p<0.001, p<0.01, respectively). These expression was significantly abolished by Avastin injection (Crush+ MF+ Avastin) (p<0.01, p<0.05, respectively) (Figure 6A-C). The ELISA analysis also the same trends (Table 3). In addition, the restoration of denervated muscle morphology in the expression of Acetylcholine and Desmin were in line with the trend of increased nerve regeneration (Figure 7A-C).<br><br>In H &E analysis, the muscle flap showed the integration of nerve related to muscle flap. There were remarkably increased vessel structure among the intra-neural structure in Crush+MF group as compared to crush group and the phenomena of increased vessel structure attenuated by Avastin treatment (Crush+ MF+ Avastin group) (Figure 8).	
Adverse Events	17	There was no adverse events	
DISCUSSION			
Interpretation / Scientific implication	18	Increase angiogenesis of crushed nerve by the rotation muscle flap is a crucial factor for nerve regeneration either in clinical or animal study. The integration of injured nerve to rotational muscle flap showed the ingrowth of vessels from the muscle and leads to increased expression of the associated angiogenesis factors. The abolishment of the angiogenesis effect by intra-muscular Avastin injection caused the reciprocal effect and furthermore confirmed this hypothesis. In addition, there was no adverse effect of increased pain sensory	Page 15-18

		threshold after muscle flap rotation. Thus, the strategy of muscle flap rotation in the assistance of nerve regeneration could be a treatment option in nerve repair.<br><br>Endogenous angiogenesis is a biological process responded to the injury in which new vessels are formed from old capillaries and the integration of nerves and vessels constitute a complicated branching network within the injured nerve (6, 7).Neurobehavior, gastrocnemius muscle mass and morphometric indices confirmed a faster recovery of regenerated axons in VEGF administration. In Immunohistochemical assessment, reactions to S-100 in VEGF group were more positive than that in silicone group (15). Local application of VEGF promotes the invasion of Schwann cells and neovascularization, which are important during nerve regeneration (16). Thus, either endogenous or exogenous supplement of angiogenesis factor were essential for nerve regeneration and this further confirmed our hypothesis.<br><br>Avastin was an immunoglobulin G monoclonal antibody directed against VEGF either in the treatment of cancer or aged-related macular degeneration (27, 19). In this study, rotational muscle augmented the increased vascular structure over the injured nerve and the associated angiogenesis factors. As the same the dosage used in macular degeneration (19), intramuscular injection of Avastin attenuates the microvascular structure and angiogenesis factors in the crushed nerve. The up and down regulation of angiogenesis factors in the crushed nerve paralleled the increased or decreased nerve regeneration were demonstrated in this study and the results highlight the effects of angiogenesis contributed to nerve regeneration.<br><br>There is still a debate concerning the angiogenesis beneficial or detrimental for nerve degeneration. Accumulating evidence suggests that pathological angiogenesis caused by the VEGF cascade in the inflammatory state is regulated by circulating leukocytes (28). Angiogenic properties of macrophages and neutrophils are stimulated by chemokines and the impact of circulating neutrophils and macro-phages in angiogenesis was contributed to the development of neuropathic pain (29, 30). In this study, the increased angiogenesis of nerve by the muscle flap rotation did not promote the recruitment of inflammatory cells (data not shown) involved in inflammatory response. Furthermore, there was also no increase in nociceptor sensation after angiogenesis by the muscle flap rotation. Thus, the rotational flap did not increases the expression of inflammatory response in the injured nerve which contributed to angiogenesis involved in the neuropathic pain.	
Generalisability/<br>Translaction	19	This study showed that the muscle flap rotation augment the angiogenesis of injured nerve. These increased microvascular structure was in line with the nerve regeneration and neurological outcome. Thus, the combined neurolysis and rotation of muscle flap to wrap the injured nerve seem to be an adjuvant therapy for nerve repair.	Page 18

Funding	20	The funding (TCVGH-1074904C and TCVGH-PU1078101) was received from Taichung Veterans General Hospital, Taichung, Taiwan	In title page

References:
1. Kilkenny C, Browne WJ, Cuthill IC, Emerson M, Altman DG (2010) Improving Bioscience Research Reporting: The ARRIVE Guidelines for Reporting Animal Research. *PLoS Biol* 8(6): e1000412. doi:10.1371/journal.pbio.1000412
2. Schulz KF, Altman DG, Moher D, the CONSORT Group (2010) CONSORT 2010 Statement: updated guidelines for reporting parallel group randomised trials. *BMJ* 340:c332.
