# Supplementary figures and images for: Increased angiogenesis by the rotational muscle flap is crucial for nerve regeneration
Source: PLoS One. 2019 Jun 10;14(6):e0217402. doi: 10.1371/journal.pone.0217402 (PMC6557495; doi:10.1371/journal.pone.0217402)

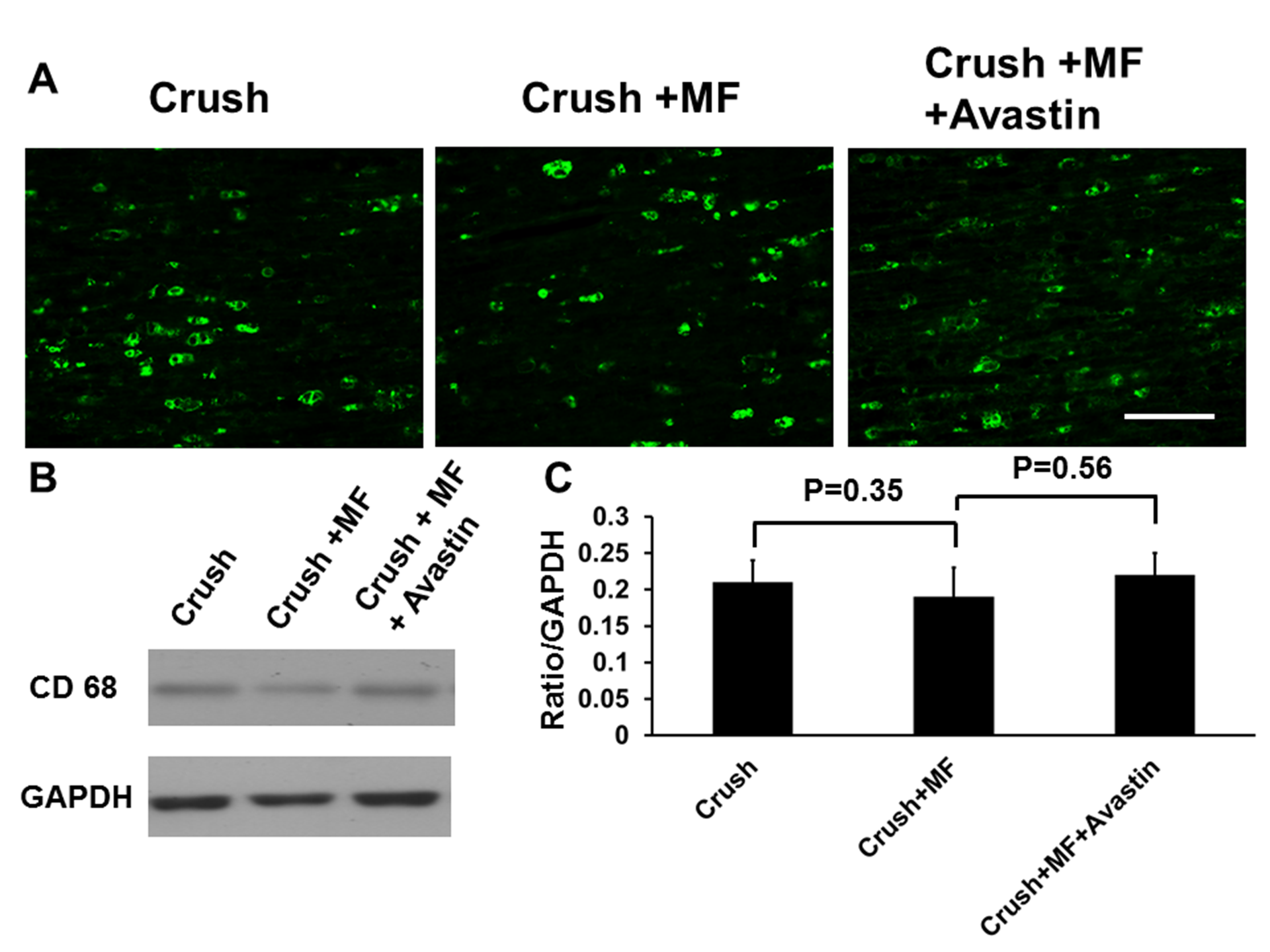

Supplement: S1 Fig — (A) The illustration of CD 68 distributed in crushed nerve subjected to various treatments. (B) A representative of western blot showing expression of CD 68 in the various treatment groups. (C) Quantitative analysis of CD 68 in the various treatment groups. Crush, Crush+MF, Crush+MF+Avastin: see text. Arrow bar length = 100 μm. (TIF) [file pone.0217402.s002.tif]
